# “Superheroes? No, thanks.” Accepting vulnerability in healthcare professionals

**DOI:** 10.1186/s12960-024-00899-9

**Published:** 2024-02-20

**Authors:** Dolores Morera, Janet Delgado, Elena Lorenzo, María Elisa de Castro-Peraza, Naira Delgado

**Affiliations:** 1 Departamento de Psicología Cognitiva, Social y Organizacional, Facultad de Psicología y Logopedia, Universidad de La Laguna, Campus de Guajara, 38205 La Laguna, Tenerife (Canarias) Spain; 2 Departamento de Filosofía I, Facultad de Filosofía y Letras, Universidad de Granada, Campus Universitario de Cartuja, 18071 Granada (Granada), Spain; 3 Escuela de Enfermería Nuestra Señora de Candelaria, Ctra. Gral. del Rosario, 145, 38010 Santa Cruz de Tenerife, Tenerife (Canarias) Spain; 4 Instituto Universitario de Neurociencia (IUNE), Universidad de La Laguna, Campus de Guajara, 38205 La Laguna, Tenerife (Canarias) Spain

**Keywords:** Stigma, Burnout, Healthcare, Vulnerability

## Abstract

In this commentary, we develop a conceptual proposal aimed to explain why a discourse of praise and admiration for healthcare professionals´ limitless dedication can trigger a general indifference to the burnout and suffering they experience. Ultimately, this can lead to the justification of the lack of resources dedicated to preventing these problems. We first start by pointing out the stigmatisation of healthcare professionals suffering from burnout and showing their vulnerability, highlighting the complex interactions that occur in the healthcare context and that increase the risk of perpetuating their suffering. Then, we appeal to the recognition of one’s own vulnerability as a key element towards the creation of a culture more focused on the duty of care for those who care for others. We conclude with several proposals for action to cope with burnout-related stigma, trying to change the superhuman image of health personnel and incorporating the vulnerability inherent to human beings.

## Introduction

As a result of the Covid-19 pandemic, roughly 115,500 healthcare professionals (HCPs) died between January 2020 and May 2021 [1], a number that does not account for deaths secondary to individual and occupational stressors. They reflect in part the failure of international governments and healthcare leaderships to prioritise the health and safety of HCPs. The tolerance of society, governments, and national health systems in subjecting health personnel to such harsh conditions, without sufficiently reinforcing physical and human resources during all this time is, to say the least, shaking.

During the pandemic, the public discourse about HCPs was elaborated in terms of “fight”, “battle”, and “heroes”. This type of narrative in the media highlights a social image of heroism that had a negative counterpart for health workers. Even when based on admiration and appearing to be a positive form of social perception, superhumanisation has negative effects [2], reducing the perception of these persons as capable of experiencing pain. This is relevant because failure to recognise someone’s pain reduces empathy and justifies refusing aid when it is needed.

The aim of this contribution is to rationalise and make visible the risks hidden behind a discourse of praise and admiration for the HCPs’ limitless dedication, and the consequent need to recognise HCPs’ vulnerability as a key aspect of their professional role. We first start by pointing out the stigmatisation of HCPs who suffer burnout, highlighting the complex interactions that occur within the healthcare context and that increase the risk of perpetuating suffering in HCPs. Then, we claim the recognition of vulnerability as a key element towards a culture more focused on the duty of care for those who care for others. We will conclude with several proposals for action aimed at coping with HCPs burnout-related stigma by changing the superhuman image of health personnel and incorporating the concept of vulnerability which is inherent to human beings.

## Suffering in silence: burnout-related stigma leads to isolation

The idea of vulnerability, fragility, fear, or any hint of weakness are outside the definition of the prototypical role of health personnel, being socially misunderstood and even punished. The manifestation of any of them leads to pointing out and stigmatising those who present it. Actions aimed at protecting and improving the well-being of professionals have overemphasised the notion of personal resilience, placing the burden of managing emotional distress solely on individual clinicians [3]. This tendency aggravates it considerably and generates more suffering and feelings of loneliness, or what has been called suffering in silence [4]. In this context, feelings of being stigmatised emerge.

Stigmatisation is a complex and dynamic process that involves both the stigmatised person and their social environment [5]. In this sense, the expression of one’s own vulnerability in the health context can imply a set of negative and blaming attitudes and responses on the part of the system and the organisation, which can also negatively transform the affected professional´s own view. These professionals not only know the rejection that any sign of mental distress provokes in their colleagues (anticipated stigma), but they also reject the distress they feel themselves (internalised stigma). Therefore, instead of seeking help and social support, they tend to hide these signs of vulnerability, which contributes to aggravating the problem.

**Fig. 1:**
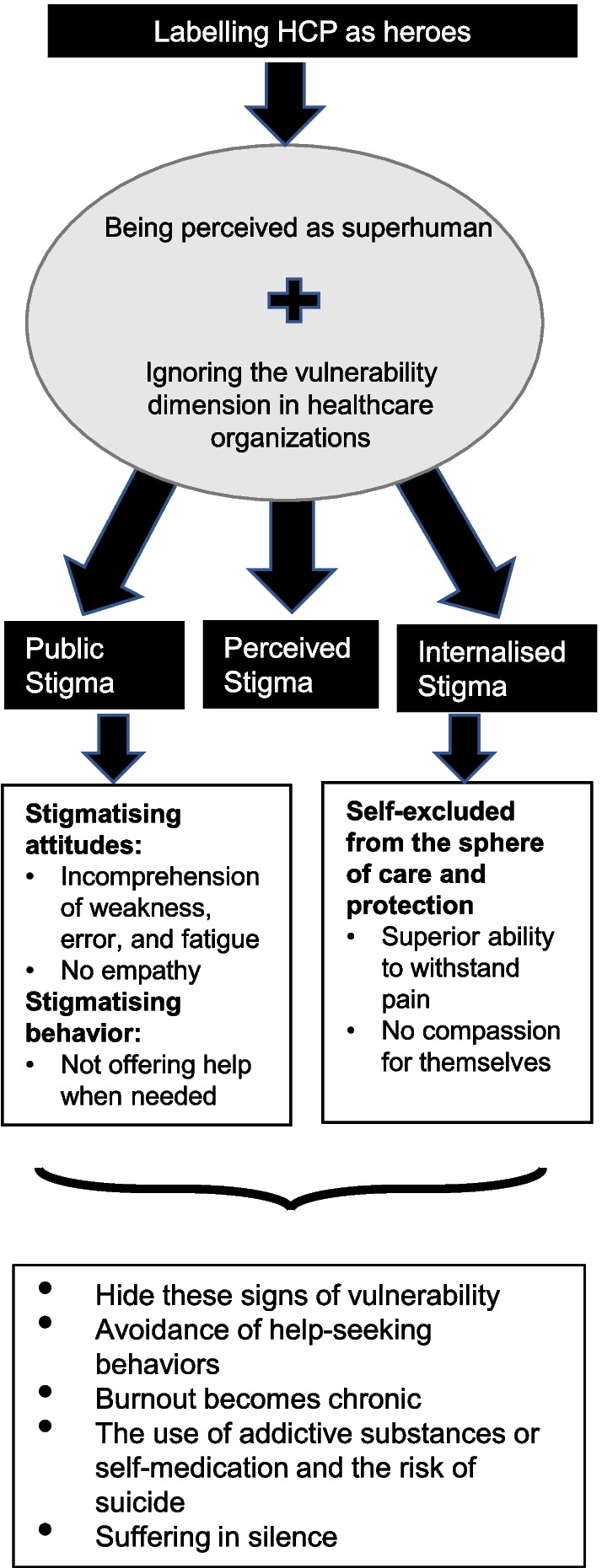
Consequences of labelling healthcare professionals as heroes

In the healthcare context, stigma is reinforced within a medical culture that promulgates high expectations, emphasising the belief that self-sufficiency, self-sacrifice, physical and emotional exhaustion are part of the professional identity. Attending one’s own needs and self-care can be seen as selfish, and help-seeking as a sign of weakness [6]. People who socialise in this context internalise these values, which turn against themselves when they begin to feel their own vulnerability in such a physically and emotionally demanding context as healthcare organisations. Figure 1 represents the negative impact for HCPs of being labelled as “superheroes”.

Approaching the problem of burnout as a process of social stigmatisation provides a new angle of analysis and, therefore, new opportunities for intervention. First, it makes visible a phenomenon that has been minimised and hidden, with severe effects for HCPs. Second, by considering a complex psychosocial process acting on multiple directions and levels (professionals, patients, and system), in line with the conceptualisation of the problem from a single new term that can produce the impact necessary to implement solutions. Since approaching the problem from the concept of well-being, a positive term, does not alert us to the urgency of addressing the suffering and discomfort experienced by health workers, the term stigmatisation could lead to focus on the social environment that influences the HCPs. Third, as stigma is the main barrier to seeking professional help, reducing the stigmatisation process could help to discover and understand the serious consequences of this phenomenon. In an anonymous survey of 1,048 academic physicians (response rate 40%), stigma and access to treatment were major concerns related to seeking treatment for a mental health concern [4, 6]. This may then result in a double-edged problem [7]: Burnout prevalence grows (as the likelihood of self-labelling increases), while the likelihood of help-seeking behaviours decreases (thus preventing appropriate treatment).

## Recognising vulnerability as a key element to overcome the burnout-related stigma

We claim that burnout-related stigma can be prevented by recognising the inherent vulnerability of HCPs—and positively considering it—as part of the professionals´ human—not superhuman—distinctiveness. Based on this recognition, preventive actions can be implemented at the organisational level. Vulnerability is a fundamental aspect in healthcare [8]: HCPs are exposed to witness fragility, suffering, pain, and death in their professional lives on a regular basis. Particularly, nursing staff may be prone to more than ordinary vulnerability, as they are routinely exposed to stressors that most people do not encounter in their everyday life. All this entails an almost daily reminder of the fragility of life and human goods [9]. While caring for patients and their families, HCPs may also suffer, since they share and reflect on the fear, anguish, and sadness that accompany these interactions [10]. Failure to recognise this intrinsic vulnerability in the performance of the profession can affect both healthcare personnel as well as patients and their families. These experiences may increase the risk of burnout, moral distress, and compassion fatigue, especially if HCPs are involved in a non-supportive work environment [11].

Vulnerability can be understood as the possibility of being harmed. It also is an inevitable part of the human condition, and it can offer a unifying foundation for a more equitable and just society. Although all people are constitutively vulnerable, the degree of exposure to certain hazards may be determined by the conditions of their particular situation. In the context of healthcare, the vulnerability of HCPs that arises from being exposed to others’ suffering and pain on a regular basis can be associated with compassion fatigue, medical errors, moral suffering, or the fear of the stigma of being affected by any of them (see Table 1 for clarifying related concepts).

**Table 1:** Other terms related to the HCPs’ vulnerability

Related terms	Definition	Characteristics
Compassion fatigue (CF)	The stress resulting from exposure to a traumatised individual or a person suffering, rather than from exposure to the trauma or suffering itself (Figley, 1995). HCPs or caregivers can develop an extreme state of tension and preoccupation with the emotional pain and/or physical distress of those being helped (Cocker and Joss 2016)	Exhaustion, anger and irritability, negative coping behaviours, reduced ability to feel empathy, decreased satisfaction with work, increased absenteeism, and less ability to make decisions and attention to patients
Medical errors	The hierarchical culture of healthcare keeps the stigma about mistakes and failures, and this remains a barrier to sharing dilemmas (Delgado et al. 2020) In 2000, To Err Is Human (Institute of Medicine, 2000) was the first publication aimed at breaking the silence that has surrounded medical errors and their consequences. The authors stated that the problem was not the fact that bad healthcare professionals were working, but rather than good HCPs were working in bad systems that needed to be made safer	Feelings of guilt, fear, shame (related to their reputation, their job, their license, and their own future as well as that of their patient), and isolation. Unwillingness to talk to anyone about the event, inhibiting both their learning and the likelihood of achieving resolution, which compounds the harm (Delbanco and Bell 2007)
Moral suffering	The anguish that HCPs and caregivers may experience in the face of moral damages, or moral residues, which can harm their integrity (Rushton, 2018). Moral suffering is inherent to healthcare practice, and it can be manifested as vulnerability, moral distress, and sometimes burnout (Delgado et al. 2020)	There are various types of moral suffering arising from diverse sources: witnessing, participating in, or directly precipitating situations that produce a wide range of negative moral outcomes, and that can trigger moral distress (Rushton, 2018). Any type of moral suffering can threaten HCPs’ integrity and can contribute to disengagement and alienation from their professions, the patients being helped, their organisations, and themselves
Fear of stigma	Fear of stigma refers to people’s constant vigilance toward the possibility of stigma and involves an inherent emotional component (Whitley and Campbell 2014). Stigma can be perceived as a major potential problem, which can even affect licence and future career. This awareness of the ways in which stigma can be harmful to oneself can lead to the ongoing fear of being stigmatised (Benz et al. 2021)	Vigilance toward stigma leads to feelings of fear, guilt, and shame about being judged by others, as well as self-rejection and a reluctance to admit that one has such problems. Fear of stigma may be a primary factor in avoiding seeking mental health treatment when necessary (Brower 2021; Clough et al. 2019)

Recognising and accepting vulnerability can have positive and generative elements [9, 12]. Expressing vulnerability can help people engage with and care for one another in a more meaningful way, to cope with difficult situations [13]. It can trigger feelings of empathy and motivate action, improving human warmth, respect, and care. Furthermore, it can help professionals to recognise the commonalities that they share with other team members, increasing the connection with their colleagues and developing a more respectful and non-critical attitude towards them. Through this awareness, HCPs normalise their own sense of vulnerability and reduce the negative feelings associated to being vulnerable.

## Challenges to overcome: a call to action

How can we improve the situation? How can we contribute to breaking this problematic conceptualisation of HCPs’ suffering? Undoubtedly, there is much to do. We propose some clues that serve as a guide to develop future interventions based on the exposed conceptual model.

The first challenge consists of *starting to identify vulnerability as a common human trait*, which allows this notion to be dissociated from that of mental illness. This “depathologisation” could help to reduce the stress and guilt felt by professionals when they experience negative emotions in the work context, as it is a natural and adaptive response to the system. Going beyond, recognising vulnerability as a normal process could lead to transforming the common definition and professional identity, including the concept of vulnerability as an inherent characteristic of the medical profession.

A second important clue is *changing the focus from an individual problem to a complex social reality*. In this way, emotional stressors should be conceived as occupational hazards instead of mental health problems [3]. The system and organisations must prioritise the care of its personnel, precisely because they are vulnerable. And that vulnerability is in turn a guarantee for the proper functioning of the system and the service to patients. Therefore, it is necessary to reduce the stigmatisation of vulnerability by recognising it, expressing it, and appreciating it as a source of experience for personal and professional improvement. The notion that sensitivity and suffering in the face of the suffering of others drives to prosocial behaviour and positive practices must be highlighted by organisations.

A third clue includes working to *enable people or groups experiencing suffering and burnout to express their own needs and provide them with social and organisational support.* The notion of self-disclosure of one’s own emotions, worries, and suffering would lead to beneficial psychological effects [14]. However, there must be an adequate institutional context to prevent adverse consequences for the person who opens up. Health institutions could create a positive organisational climate to understand and support other colleagues in their vulnerability. Understanding the distress faced by healthcare workers during times of crisis, considering it in general as part of the work, and accepting this vulnerability can have positive effects and help to reduce the perception of loneliness, or what has been called suffering in silence [4].

*Improving social connections for sharing emotions* could be a key element to reduce barriers to engage in interventions aimed at supporting healthcare workers [11]. Specific interventions have been implemented for health professionals to reflect together on the emotional impact of their work. Multidisciplinary forums have obtained results in improving the well-being of professionals and have also contributed to a change in the medical culture. It seems that the disclosure through narration that occurs in this type of activity offers the possibility for health professionals to show vulnerability [14]. Notice that the conditions of the group (size, ethical climate, goals, etc.) must be optimised for guarantee the effectiveness of this type of interventions. In this line, interventions based on the theoretical model of the effects of sharing emotions [15] could be especially useful, highlighting that emotional recovery requires fundamentally cognitive work related to goals and meaning instead isolated emotional work.

Finally, the challenge of *expanding the framework of shared responsibility and the need for system-level solutions* should be assumed by healthcare organisations. Despite declarations that clinicians’ well-being is an organisational priority, support programmes are often poorly resourced, and leaders are rarely held accountable for outcomes related to well-being. Even if perpetuating the status quo and ignoring these barriers may appear to be unoffensive, lack of attention to well-being is ultimately extremely costly. Mistrust in organisations also keeps some clinicians from seeking help, since medical institutions have historically punished clinicians who have mental health issues. Other factors have further eroded clinicians’ trust that their organisations will support them, such as a pattern of valuing productivity over well-being and a failure to address health care disparities that have been highlighted during the pandemic. In this sense, an unequivocal, stable, and resistant commitment over time is required for health institutions. The support for health institutions could be particularly complex in settings where resources are scarce, and challenges are overwhelming. In these environments, healthcare workers face a unique combination of challenges, including the lack of basic medical equipment, shortages of trained personnel, and limited access to quality healthcare services. Understanding the context in which these professionals operate is crucial for designing more effective interventions and advocating for structural changes that improve working conditions and healthcare delivery in the most underserved communities.

## Conclusions

Although their positive intention, messages depicting clinicians as heroes imply a high expectancy of personal sacrifice without considering their own suffering or personal costs [3]. These cultural frames of reference, linked with the lack of integration of vulnerability as a part of the role of HCPs, lead to institutional ostracism and stigmatisation. HCPs tend to feel alone and guilty in their vulnerability and suffering, convinced that other colleagues are successfully handling these situations. In this sense, the culture of medicine and the socialisation process in healthcare organisations play a relevant role. Any help-seeking behaviour could be perceived as a sign of weakness [4, 6], receiving a blatant or subtle kind of social punishment. Without help and support, suffering, burnout, turnover intentions, and suicidal thoughts become unstoppable.

For many years, the culture of healthcare organisations has been dominated by silence and avoidance of healthcare professionals’ own feelings and emotions. This culture has been reinforced during the Covid-19 pandemic under the idea that healthcare professionals are heroes that can face any terrible situation, regardless how hard it is or the cost that it can have for their personal life: “at the end, they are heroes”. This collective cultural vision of health professionals can increase the potential harm to which they are exposed. This commentary invites to change the culture of superheroes into a culture of human beings who are also vulnerable and who also need care. To really address burnout as a structural problem, it is crucial to create a professional identity based on the humanity of health personnel. We need to move towards a culture that can teach that vulnerability is a valuable trait of human nature because it makes us better caregivers. Undoubtedly, to change the problem it is crucial to promote the practice of the care and compassion from the organisation towards the staff, as well as care, attention, and social support for fellow caregivers.

## Data Availability

Not applicable.
